# Verification of the interaction between human bitter taste receptor T2R46 and polyphenols; Computational chemistry approach

**DOI:** 10.1016/j.crfs.2024.100914

**Published:** 2024-11-05

**Authors:** Takafumi Shimizu, Taiki Fushimi, Rio Ohno, Fujii Yasuyuki, Kenta Aso, Ursula M. Jacobs, Osamu Nureki, Yoshitomo Suhara, Vittorio Calabrese, Naomi Osakabe

**Affiliations:** a Systems Engineering and Science, Graduate School of Engineering and Science, Shibaura Institute of Technology, Japan; b Functional Control Systems, Graduate School of Engineering and Science, Shibaura Institute of Technology, Japan; c Department of Bioscience and Engineering, Faculty of System Science and Engineering, Shibaura Institute of Technology, Japan; d SIT Research Laboratories, Shibaura Institute of Technology, Japan; e Central Research Institute, ITO EN, Ltd., Japan; f System Biologie AG, Wollerau, (CH), Switzerland; g Department of Biological Sciences, Graduate School of Science, The University of Tokyo, Japan; h Department of Biomedical and Biotechnological Sciences, University of Catania, Italy

**Keywords:** Polyphenol, T2R46, Molecular docking simulation, Quantitative structure-activation relationship

## Abstract

Recent studies have indicated that the activation of bitter taste receptors (T2R) expressed in gastrointestinal secretory cells has a regulatory effect on the secretion of gastrointestinal hormones. Polyphenols are known to be ingested at a daily intake of 5 g or more and commonly have a bitter taste. Consequently, the interaction between the bitter taste receptor T2R46 and 490 polyphenols was investigated using *in silico* simulation techniques. It was demonstrated that W88^3.32^ and E265^7.39^ play a pivotal role in the recognition of polyphenols and known ligands by T2R46, with frequent interactions observed, particularly with flavonoids. The results of the quantitative structure-activity relationship (QSAR) analysis demonstrated a high degree of correlation (R^2^ = 0.9359) between polyphenols and T2R46 in a model that incorporated molecular interaction field regions and branching scales. Furthermore, known ligands were also found to fit this model (R^2^ = 0.9155). These findings suggest that polyphenols may act as T2R46 ligands.

## Highlights

• Interactions between T2R46 and 490 polyphenols were investigated *in silico*.
• Molecular docking suggests that most polyphenols bind to T2R46.
• Many flavonoids showed similar interactions to well-known T2R46 ligands.
• QSAR analysis suggested relationships between binding energy score and molecular structure.
• Interaction field area and molecular branching are crucial for T2R46 binding.

## Graphical abstract

**Figure:**
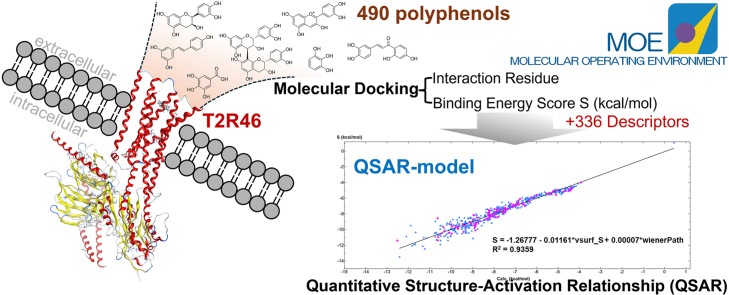

## Abbreviations used

T2R: bitter taste receptor
T2R46: bitter taste receptor member 46
QSAR: Quantitative structure-activation relationship
GPCR: G protein-coupled receptor
GI: Gastrointestinal
CCK: Cholecystokinin
GLP-1: Glucagon-like peptide-1
MOE: Molecular Operating Environment
GBVI/WSA ΔG: Generalized-Born Volume Integral/Weighted Surface area ΔG
LOO q^2^: Leave-one-out cross validated R^2^

## 1 Introduction

Type 2 taste receptors (T2Rs), which are located in the taste cells of the tongue, are responsible for detecting compounds and triggering the sensation of bitterness(Andres-Barquin and Conte, 2004; Trius-Soler and Moreno, 2024). The human T2Rs are a 291–334 amino acids long G protein-coupled receptor (GPCR) family comprising 25 to 26 members (Chandrashekar et al., 2000; Lang et al., 2023; Liu et al., 2018; Luo et al., 2019; Meyerhof et al., 2010). T2Rs are known to be expressed throughout the body, not only in the taste-sensing oral tissues. T2R46, the target of this study, is abundantly expressed in the stomach and skeletal muscle (Descamps-Solà et al., 2023; Talmon et al., 2023), and its expression has also been confirmed in the jejunum (Liszt et al., 2022; Ziegler et al., 2023) and colon(Rozengurt et al., 2006). Recently, attention has been focused on T2R expressed in gastrointestinal secretory cells. This is because gastrointestinal (GI) hormones, namely cholecystokinin (CCK) and glucagon-like peptide-1 (GLP-1), are secreted in the response to T2R activation(Chen et al., 2006; Chou, 2021; Dotson et al., 2008a, 2010b). GI hormones have beneficial effects, including the regulation of the gastric function and blood sugar levels. Polyphenols represent diverse group of secondary metabolites found in fruits, vegetables, tea, red wine, and various other plant-derived products. These compounds consist of multiple hydroxyl groups on aromatic rings, with over 8000 identified to date (Arts and Hollman, 2005; Pandey and Rizvi, 2009). Previous epidemiological and intervention studies have suggested a negative correlation between polyphenol intake and the risk of diabetes (Osakabe et al., 2024; Osakabe et al., 2024).

A number of *in vitro* studies have reported that many polyphenols have the potential to activate several T2Rs (Lossow et al., 2016; Roland et al., 2011; Soares et al., 2013, 2018).Furthermore, numerous animal studies and intervention trials have shown that polyphenol-rich fractions from various fruits and vegetables have a preferential effect on reducing fasting and postprandial blood glucose levels (Osakabe et al., 2024; Osakabe et al., 2024; Tarragon and Moreno, 2020). It is well established that polyphenols have extremely poor bioavailability, which makes it highly unlikely for them to be distributed in the bloodstream (Manach et al., 2005). These results collectively indicated that polyphenols may have facilitated the secretion of incretins such as GLP-1 by activating T2Rs, which are expressed on enteroendocrine cells in the intestinal tract. Despite the potential benefits of polyphenols, such as improved glucose tolerance, there have been no systematic studies on the interactions between the various types of polyphenols and the 25–26 T2Rs expressed in humans due to the large number of combinations. It is therefore important to examine these interactions to elucidate the beneficial effects of polyphenols (Trius-Soler and Moreno, 2024).

The present study aimed to elucidate the interaction between T2R46, and 490 polyphenols. This was achieved with *in silico* simulation methods, given that T2R46 is the first member of the T2R family for which a detailed three-dimensional structure has been revealed (Xu et al., 2022). Furthermore, quantitative structure-activity relationship (QSAR) analysis was conducted using the obtained data to identify the physicochemical properties of polyphenols that are crucial for interaction with T2R46.

## 2 Materials and methods

### 2.1 Materials

Molecular docking and QSAR were performed using Molecular Operating Environment (MOE) (Vilar et al., 2008). The 3D structure of T2R46 and test chemicals using this study were loaded into MOE. T2R46 is the human bitter taste receptor that responds to a broad spectrum of bitter substances (Brockhoff et al., 2007; Luo et al., 2019). Several studies have shown that T2R46 is activated by bitter substances and promotes GLP-1 secretion(D'Urso and Drago, 2021; Wang et al., 2020; Xie et al., 2018). In addition, Xu et al. revealed the detailed three-dimensional structure of T2R46 in September 2022 using the cryo-electron microscopy (Xu et al., 2022). Strychnine, a toxic alkaloid with a bitter taste, has been identified as the most potent agonist of T2R46 to date (Born et al., 2013; Brockhoff et al., 2007a, 2010b).

The three-dimensional structure of T2R46 was obtained as a complex sample using strychnine as the non-polyphenolic positive control. As polyphenols, 489 polyphenols with SMILES notation listed in Phenol-Explorer (http://phenol-explorer.eu/) were used (Neveu et al., 2010). These include tangeretin and nobiletin, which have been shown by *in vitro* studies to activate T2R46 (Kuroda et al., 2016). Furthermore, amarogentin, which has been demonstrated to stimulate T2R46 *in vitro*^2)^, was incorporated into the test regimen for 491 compounds (490 polyphenols and strychnine). The complete list of test polyphenols employed is provided in Supplementary Table S1.

### 2.2 Molecular docking

**Fig. 1:**
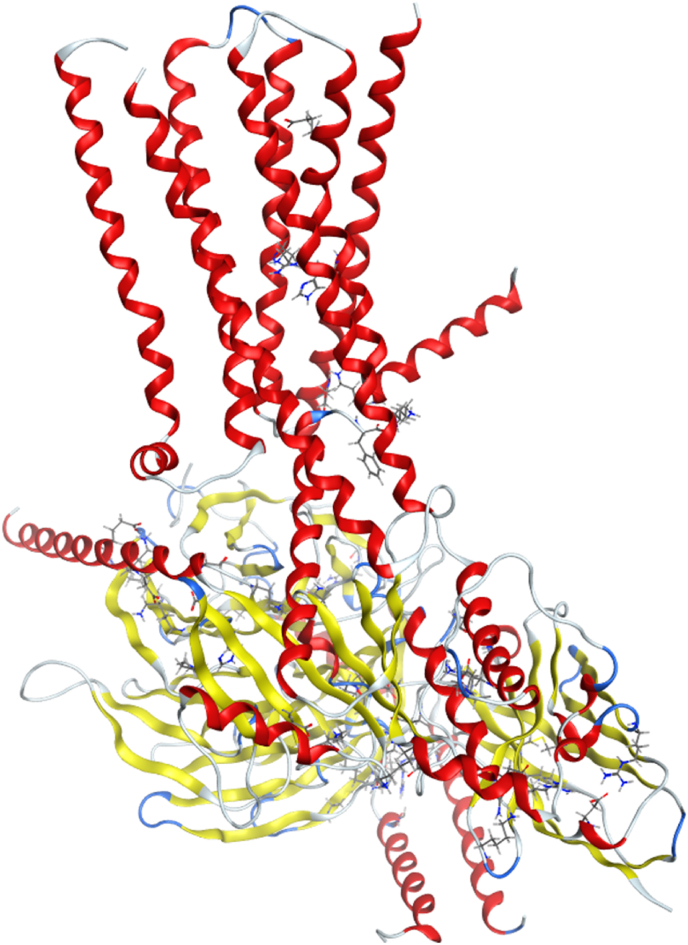
Prepared structure of T2R46 (PDB code: 7XP6; cryo-EM structure of a class T GPCR in active state) using MOE. Following the acquisition of the 3D structure from the PDB, any structural issues (e.g., broken bonds or missing loops) were addressed using the Quick Prep function of MOE.

The three-dimensional structure of T2R46 (PDB code: 7XP6; cryo-EM structure of a class T GPCR in active state) was obtained from the Protein Data Bank (PDB; https://www.rcsb.org). The structure was prepared using MOE to correct structural problems (such as broken bonds or missing loops), add hydrogens and calculate partial charges. See Supplementary Methods for details on structure preparation. Fig. 1 shows the structure of T2R46. As for the binding site of the test compounds, previous studies have shown that the ligand of T2R46, strychnine, binds from the extracellular region(Brockhoff et al., 2010; Sandal et al., 2015). Given that previous study has identified the binding site for strychnine on T2R46 (Xu et al., 2022) we have designated this site as the binding site for the test compound in the present docking. Other potential binding sites were also explored in this study, but all of them were in sites other than the T2R46 (Supplementary Methods).

**Fig. 2:**
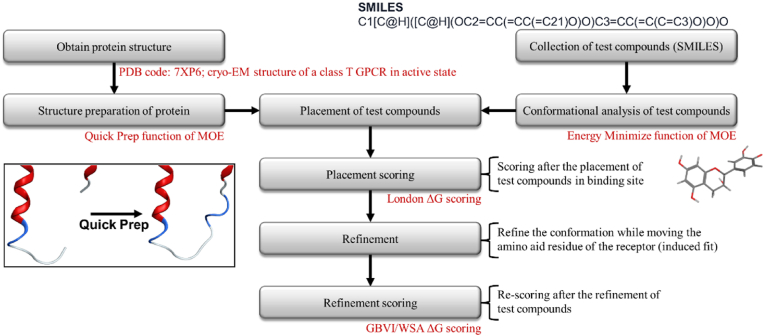
Scheme for molecular docking method. For all test compounds, alpha PMI was employed, with the number of configurations set to 1000. Various conformations of the ligand were placed. The most promising 100 conformations were then selected using the London ΔG scoring method of MOE. After subsequent refinement by induced fit, the final binding energy scores were estimated by GBVI/WSA ΔG.

Firstly, alpha PMI, a rapid and optimal method for the precise placement of ligands within narrow confines, was employed to position different conformations of the ligand, with the number of placements set to 1000. The 100 most promising conformations were then selected using the MOE's London ΔG scoring method (Corbeil et al., 2012). Further details on the London ΔG scoring can be found in Supplementary Methods (Details of London ΔG scoring). This model is employed to score a vast number of ligand placements, with the binding free energy score ΔG are calculated by considering hydrogen bonding, coordination bonding, ligand's degrees of freedom, and desolvation. Subsequently, the ligand structures were subjected to further refinement utilizing the fixed receptor option, induced fit. This refinement step is an energy minimization using the conventional Amber10 with Extended Hückel Theory molecular mechanics force field. The objective was to take electronic effects into account and to choose 20 poses. The final energy (Generalized-Born Volume Integral/Weighted Surface area ΔG scoring, GBVI/WSA ΔG scoring) was estimated after the refinement step using the generalized Born solvation model (Corbeil et al., 2012). This scoring function is based on the force field, and in this model, the binding free energy is estimated from the ligand's placement. Further information on GBVI/WSA ΔG scoring is provided in the Supplementary Methods. Fig. 2 shows the stages in the molecular docking process. The final energy ΔG obtained through these processes was employed as a descriptor, the binding energy score S, for the subsequent QSAR analysis.

### 2.3 Quantitative structure-activity relationship

**Fig. 3:**
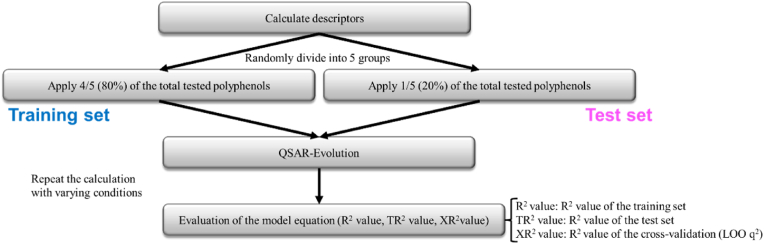
Scheme for QSAR method by using QSAR-Evolution. To avoid overfitting and to improve the prediction accuracy for new substance groups, 80% of the 490 polyphenols tested were divided randomly into a training set (392 polyphenols) and 20% into a test set (98 polyphenols).The fit for the two groups was then evaluated. Finally, the fit of the model to all polyphenols tested was evaluated, as well as the fit of the training and test sets and the results of cross-validation.

QSAR analysis was conducted to investigate the correlation between the binding energy and the structural properties of polyphenols. Initially, 336 descriptors were calculated for the polyphenols that were subjected to testing. The Supplementary Table S3 presented a comprehensive overview of the calculated descriptors and their respective classification. After calculating the descriptors for each polyphenol, the model equation was calculated utilizing QSAR-Evolution. QSAR-Evolution is a genetic algorithm that has been coded as a QSAR method, which allows for the automatic selection of descriptors. This is provided by MOE through the CCG SVL Exchange (https://svl.chemcomp.com/). Further information on QSAR-Evolution is provided in the Supplementary Methods. To avoid overfitting and to improve the prediction accuracy for new substance groups, 80% of the 490 polyphenols tested were divided randomly into a training set (392 polyphenols) and 20% into a test set (98 polyphenols). The fit for the two groups was then evaluated. Finally, the fit of the model to all polyphenols tested was evaluated, as well as the fit of the training and test sets and the results of cross-validation. Cross validation is a method for evaluating the predictive power of QSAR model (Konovalov et al., 2008), and the most popular criteria, leave-one-out cross validated R^2^ (LOO q^2^), was used as the evaluation in this study (Golbraikh and Tropsha, 2002). Fig. 3 illustrates the stages of the QSAR-Evolution process. The results obtained from this QSAR analysis were further validated. Molecular docking alongside the descriptor calculations for 68 known ligands of T2R46 listed in BitterDB (https://bitterdb.agri.huji.ac.il/dbbitter.php, Supplementary Table S4) (Dagan-Wiener et al., 2019) was performed using the same procedure, and the fit to the QSAR model obtained from 490 polyphenols was examined.

## 3 Results

### 3.1 Molecular docking

**Fig. 4:**
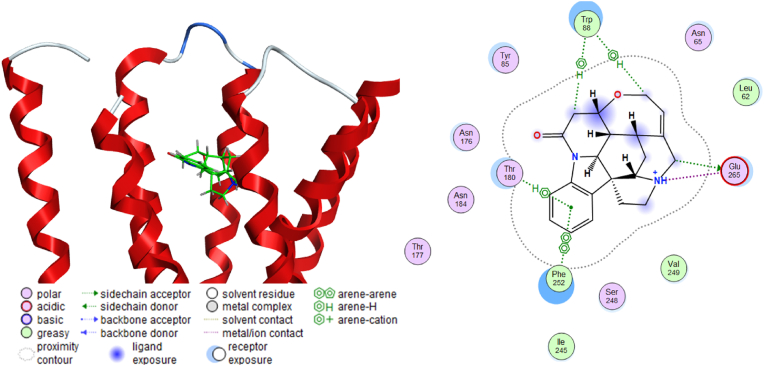
Molecular docking simulation of strychnine and T2R46. Computational scheme for the energetically lowest conformation of strychnine and T2R46. schematic of 3D (left) and 2D (right) binding interactions using MOE software. For details about the legend of the Figure on the left, please see Supplementary Fig. S3.

**Fig. 5:**
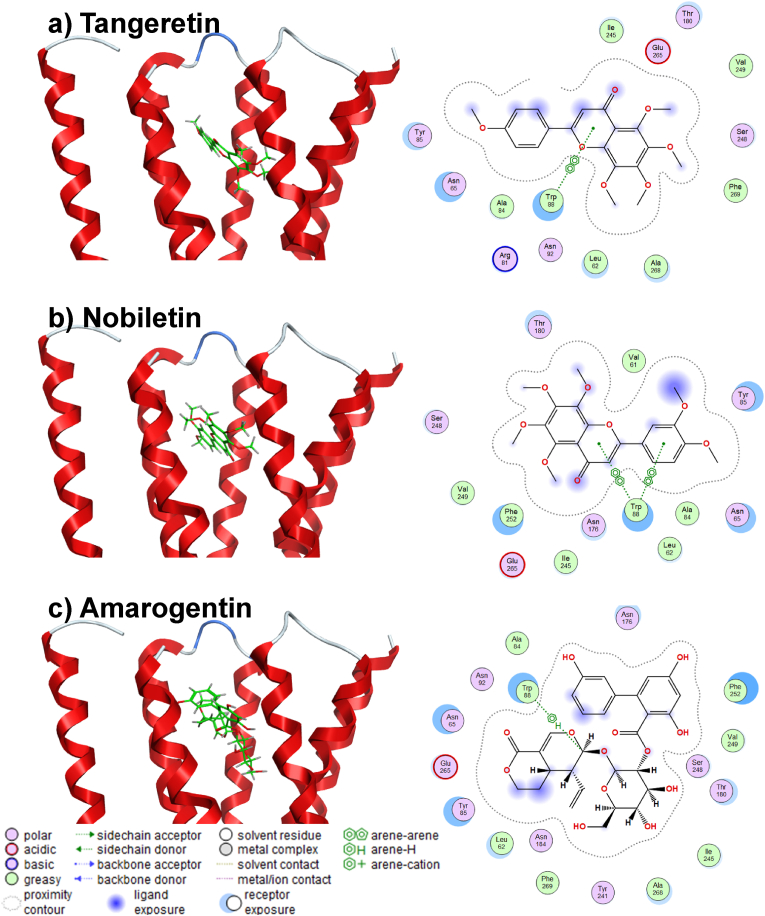
Molecular docking simulation of tangeretin, nobiletin, or amarogentin and T2R46. Diagram of the energetically lowest conformation of each polyphenol and T2R46 using MOE software. Three-dimensional (left) and two-dimensional (right) schematic binding interactions of a) tangeretin, b) nobiletin, or c) amarogentin. For details about the legend of the Figure on the left, please see Supplementary Fig. S3.

**Fig. 6:**
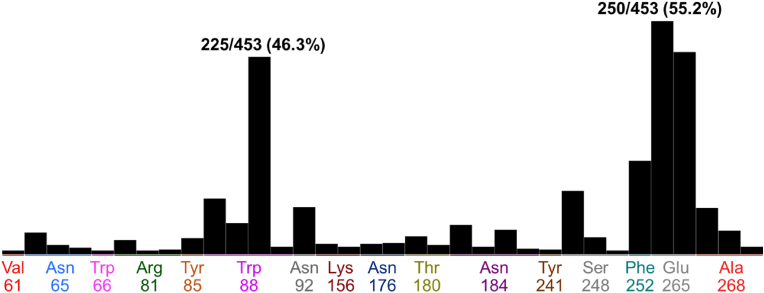
Population map of the interacting amino acid residues after extracting the results of polyphenols with the lowest binding energy score. Interactions with E265^7.39^ were the most common (250/453; 55.2%), followed by W88^3.32^ (225/453; 46.3%).

The molecular docking result of the positive control strychnine was shown in Fig. 4. This result confirmed the interaction between W88^3.36^ and E265^7.39^, which was shown to play an important role in ligand binding in a previous study (Xu et al., 2022), was similarly confirmed in this study. Furthermore, its binding energy score was −6.0767 kcal/mol. Table 1 shows the energetically lowest docking performance of strychnine along with the amino acid residue, type of interaction, distance and binding energy score for each amino acid residue in T2R46. The result of polyphenols, which have been shown in *in vitro* studies to activate T2R46; tangeretin, nobiletin (Kuroda et al., 2016), amarogentin (Meyerhof et al., 2010), were shown in Fig. 5. All polyphenols demonstrated interactions interacted with W88^3.32^, and with the binding energy scores of tangeretin, nobiletin, and amarogentin were −7.4983 kcal/mol, −7.8191 kcal/mol, and −9.4206 kcal/mol, respectively. Notably, the binding energy values of these polyphenols and strychnine with T2R46 showed a tendency to increase with decreasing molecular weight. Table 2 shows the energetically lowest docking performance of each polyphenol along with the amino acid residue, type of interaction, distance and binding energy score for each amino acid in T2R46. Fig. 6 shows the results of the interaction of polyphenols with the amino acid residues of T2R46 as the population map in the case of the conformations that exhibited the lowest binding energy values for each substance. Among all polyphenols, the interaction with E265^7.39^ was the most frequent, followed by the interaction with W88^3.32^ (55.2% and 46.3%, respectively). The percentages and details of each interaction shown in Fig. 6 are presented in Supplementary Table S5. In addition, a maximum of 20 conformations per compound were calculated in this study. The results including all conformations are shown in Supplementary Fig. S4, Supplementary Table S6. Interestingly, the conformations with the lowest binding energy values for each compound tended to interact more with W88^3.32^. As the interaction, two major features were observed in the interaction between W88^3.32^ and E265^7.39^. In the interaction with W88^3.32^, many polyphenols showed CH-π or π-π interactions between their own hydrogen atoms or aromatic moieties and the aromatic moieties of W88^3.32^. In the interaction with E265^7.39^, all polyphenols with which interactions were observed acted as hydrogen bond donors (salt bridge interaction). These interactions were observed especially in polyphenols with a flavonoid skeleton, but among the polyphenols tested, methoxyflavonoids (methoxyflavone, methoxyflavonol, methoxyflavanone), in which the OH group was replaced by a methoxy group, showed no interactions with E265^7.39^ at all (Table 3). The results of the interaction of all polyphenols with W88^3.32^ and E265^7.39^, including binding energy score, are shown in Supplementary Table S7. Table 4 shows the results for the top 10 and bottom 10 polyphenols with the lowest binding energy scores. It is important to note that the calculated binding energy values for all polyphenols, except rubusaviin B, were negative.

**Table 1:** Predicted binding energy of strychnine with amino acid residue in T2R46 by using MOE.

Compound	MW (g/mol)	Amino Acid Residue	Interaction	Distance (Å)	Binding Energy (kcal/mol)
Strychnine	334.41	GLU265	H-Donor	3.24	−1.6
		GLU265	H-Donor	3.23	−1.7
		GLU265	Ionic	3.55	−1.7
		TRP88	H-pi	3.99	−0.5
		TRP88	H-pi	4.19	−0.5
		THR180	pi-H	3.83	−0.5
		PHE252	pi-pi	3.99	−0.0

**Table 2:** Predicted binding energies of tangeretin, nobiletin, or amarogentin with amino acid residue in T2R46 by using MOE.

Compound	MW (g/mol)	Amino Acid Residue	Interaction	Distance (Å)	Binding Energy (kcal/mol)
Tangeretin	372.37	TRP88	pi-pi	3.38	−0.0
Nobiletin	402.39	TRP88	pi-pi	3.91	−0.0
		TRP88	pi-pi	3.63	−0.0
Amarogentin	586.54	TRP88	H-pi	3.78	−0.6

**Table 3:** Results of methoxyflavonoids used in this study.

Family	Compound	W88^3.32^	E265^7.39^	Binding Energy Score (kcal/mol)
Metoxyflavones	Nepetin	--R-	–	−6.5912027
Metoxyflavones	Geraldone	–	–	−6.1617451
Metoxyflavones	Pebrellin	--R-	–	−7.0225263
Metoxyflavones	Eupatorin	--R-	–	−7.2291708
Metoxyflavones	Nobiletin	--R-	–	−7.8191781
Metoxyflavones	Hispidulin	--R-	–	−6.4276075
Metoxyflavones	Gardenin B	--R-	–	−7.7319427
Metoxyflavones	Jaceosidin	--R-	–	−6.5976892
Metoxyflavones	Sinensetin	--R-	–	−7.7238488
Metoxyflavones	Tangeretin	--R-	–	−7.498385
Metoxyflavones	Cirsilineol	--R-	–	−7.0213504
Metoxyflavones	Tetramethylscutellarein	--R-	–	−7.2179251
Metoxyflavones	Cirsimaritin	--R-	–	−6.8335447
Metoxyflavones	5,6-Dihydroxy-7,8,3′,4′-tetramethoxyflavone	–	–	−7.4341669
Metoxyflavonols	Isorhamnetin	--R-	–	−6.3432798
Metoxyflavonols	3-Methoxynobiletin	--R-	–	−8.244936
Metoxyflavonols	3-Methoxysinensetin	--R-	–	−7.9135222
Metoxyflavonols	3,7-Dimethylquercetin	--R-	–	−6.6431146
Metoxyflavonols	Kaempferide	–	–	−5.9693704
Methoxyflavanones	Hesperetin	--R-	–	−6.118628
Methoxyflavanones	Sakuranetin	–	–	−5.9838963

∗, -, No interaction; R, Arene interaction (CH-π or π-π interaction).

**Table 4:** Top 10 and bottom 10 binding energy scores of polyphenols tested.

	Compound	MW (g/mol)	Binding Energy (kcal/mol)
Top 10 compounds			
	Spinacetin 3-O-(2-feruloylglucosyl)(1->6)-[apiosyl(1->2)]-glucoside	978.86	−13.496622
	2,5-di-S-Glutathionyl caftaric acid	922.85	−12.451329
	Kaempferol 3,7,4′-O-triglucoside	772.66	−12.116314
	Cinnamtannin A2	1155.04	−12.063482
	1-Sinapoyl-2,2′-diferuloylgentiobiose	900.84	−11.917153
	Kaempferol 3-O-(2″-rhamnosyl-6″-acetyl-galactoside) 7-O-rhamnoside	784.67	−11.904759
	Patuletin 3-O-(2″-feruloylglucosyl)(1->6)-[apiosyl(1->2)]-glucoside	964.83	−11.840207
	Chrysoeriol 7-O-(6″-malonyl-apiosyl-glucoside)	680.57	−11.411658
	1,2′-Disinapoyl-2-feruloylgentiobiose	930.86	−11.398185
	Cyanidin 3-O-glucosyl-rutinoside	757.67	−11.396862


Bottom 10 compounds			
	4-Methylcatechol	124.14	−4.5744896
	Benzoic acid	122.12	−4.5581765
	3-Methylcatechol	124.14	−4.5317850
	4-Vinylsyringol	120.15	−4.5034232
	4-Vinylphenol	120.15	−4.4993973
	4-Hydroxybenzaldehyde	122.12	−4.4832768
	Pyrogallol	126.11	−4.4441705
	Catechol	110.11	−4.1428356
	Phenol	94.11	−4.0015717
	Rubusuaviin B	2805.92	1.0888770

### 3.2 Quantitative structure-activity relationship (QSAR-Evolution) of test polyphenols

**Fig. 7:**
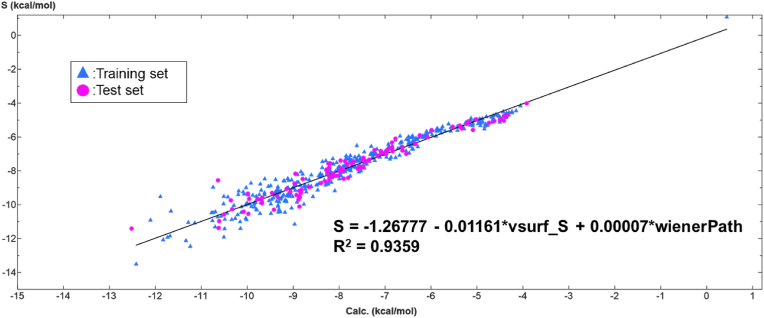
Scatter plots for the 3D-QSAR model predicted and calculated binding energy of all tested polyphenols (N = 490). The fitted equations and R^2^ values are shown as well in the corresponding plots. Calc.: binding energy score estimated by the model equation. S: binding energy score obtained by molecular docking.

The best model obtained by QSAR-Evolution follows the equation Eq. (1):

(1) S=−1.26777−0.01161∗vsurf_S+0.00007∗wienerPath

where *S* is the binding energy score, *vsurf_S* is the interaction field surface area (Cruciani et al., 2000), *wienerPath* is the Wiener path number; half the sum of all the distance matrix entries as defined in (measure of molecular branching) (Balaban, 1979; Wiener, 1947). See Supplementary Table S8 for the detailed calculation conditions under which this model was obtained. The scatter plot of the binding energy values predicted by the 3D-QSAR model and those calculated by molecular docking simulation for all polyphenols is shown in Fig. 7.

**Fig. 8:**
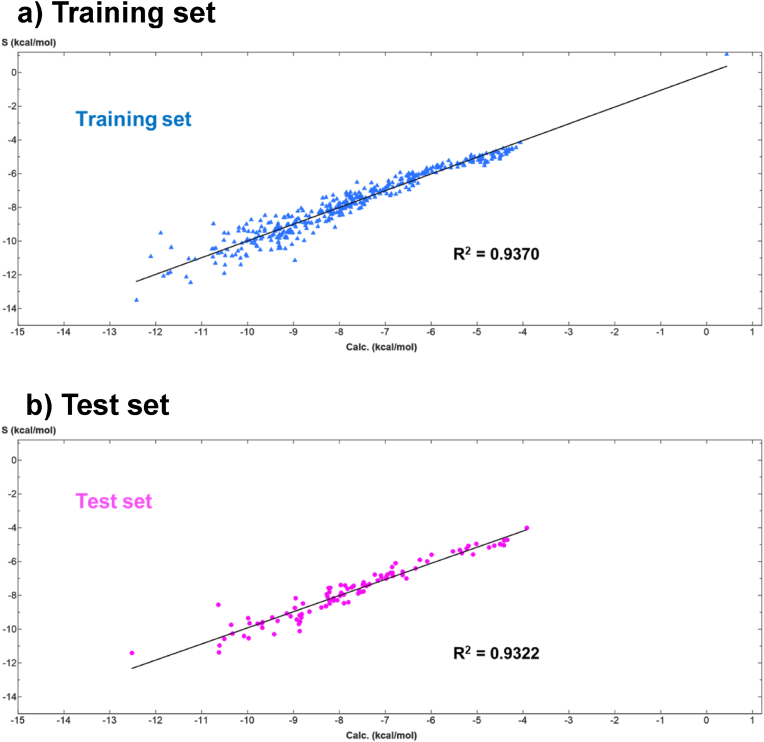
Scatter plots for the 3D-QSAR model predicted and calculated binding energy. a) training set (N = 392, 80 % of compounds), b) test set (N = 98, 20% of compounds) The fitted equations and R^2^ values are shown as well in the corresponding plots. Calc.: binding energy score estimated by the model equation. S: binding energy score obtained by molecular docking.

The model was fitted to all test polyphenols, with an R^2^ value of 0.9359. The fit was also verified for the training set (N = 392) and the test set (N = 98), with R^2^ values of 0.9370 and 0.9322, respectively (Fig. 8).

Furthermore, leave-one-out cross validated R^2^ (LOO q^2^) of this model was 0.9003.

### 3.3 Molecular docking and QSAR of non-polyphenolic bitter compounds excelled on a database

**Fig. 9:**
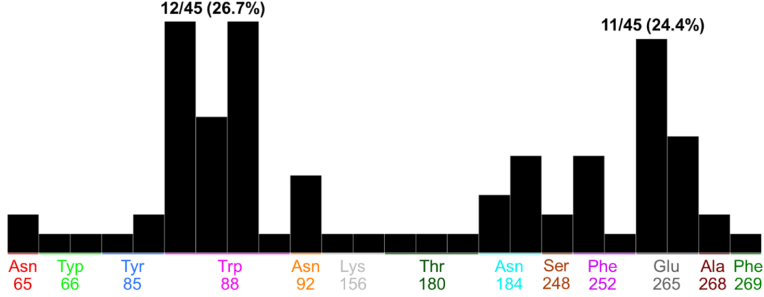
Population map of the interacting amino acid residues of known T2R46 ligands. W88^3.32^ was the most common interaction in all calculation results (12/45; 26.7%). E265^7.39^ was next (11/45; 24.4%).

**Fig. 10:**
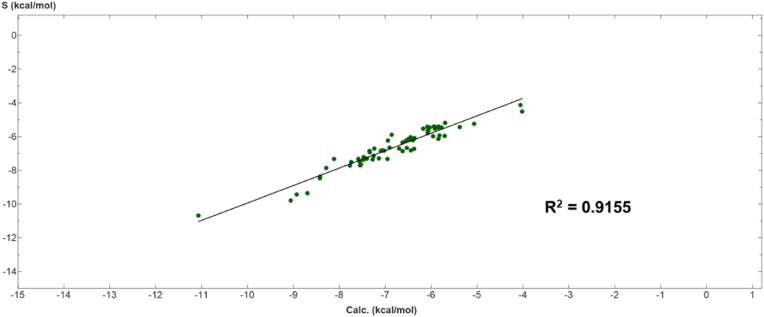
Fit of known ligands of T2R46 to the model obtained by QSAR-Evolution. Calc.: binding energy score estimated by the model equation. S: binding energy score obtained by molecular docking. After similar molecular docking of 68 known ligands of T2R46, descriptors were calculated and deduced by substituting them into the model obtained by QSAR (R^2^ = 0.9155).

Molecular simulations were performed on 68 known ligands registered in the Bitter database, employing a methodology identified for polyphenols. The amino acid residue in T2R46 that interacted most was W88^3.32^ (Fig. 9). The percentages and details of each interaction shown in Fig. 9 are presented in Supplementary Table S9. The results including all conformations are shown in Supplementary Fig. S5, Supplementary Table S10. Furthermore, the compatibility of the ligands with the model equation calculated using the results for polyphenols was verified, resulting in an R^2^ value of 0.9155 (Fig. 10). The results of molecular simulation of 68 different known ligands are shown in Supplementary Table S11.

## 4 Discussion

Molecular docking of strychnine, tangeretin, nobiletin, and amarogentin, which have been demonstrated to activate T2R46 in previous *in vitro* studies, revealed that these substances interact with the amino acid residue W88^3.32^ of T2R46 (Kuroda et al., 2016; Meyerhof et al., 2010). A comprehensive analysis of the known T2R46 ligands in the Bitter database revealed that the most frequent interaction was with the amino acid residue W88^3.32^. This finding is consistent with previous studies, which have demonstrated that the interaction between the ligand and W88^3.32^ plays an important role in T2R46 activation (Xu et al., 2022). Among the interactions observed in the polyphenols used in this study, those with W88^3.32^ and E265^7.39^ were particularly prevalent (Fig. 6). The most frequently identified forms of interaction in this study were the CH-π and π-π interactions between W88^3.32^ and the hydrogen atom or aromatic moiety of polyphenols, and the salt bridge interaction with E265^7.39^. Previous studies have shown that the therapeutic drugs quinine, berberine, and chlorpheniramine are stabilized by a combination of interactions with the aromatic moiety of W88^3.32^ and salt bridge interactions with E265^7.39^ (Vitaku et al., 2014; Xu et al., 2022). Thus, in addition to W88^3.32^, which was identified as important for T2R46 activation in this study, the salt bridge interaction with E265^7.39^ may play an important role in ligand recognition and binding stabilization. Therefore, polyphenols that showed salts bridge interaction with E265^7.39^ in addition to the CH-π and π-π interactions with W88^3.32^ are likely to activate T2R46. The prevalence of these amino acid interactions for polyphenols classified as flavonoids was particularly high in this study. These interactions were observed in 81 out of 263 polyphenols classified as flavonoids, compared to 18 out of 227 polyphenols classified as other polyphenols, suggesting that these compounds may be able to activate T2R46. However, among the test polyphenols, those classified as methoxyflavonoids showed many interactions with W88^3.32^, while no interaction at all with E265^7.39^ was observed. Previous studies have reported that methoxyflavonoids could exhibit orthosteric inhibition as antagonists of T2R, the mechanism of which is the lack of hydrogen donors (Jalševac et al., 2022; Roland et al., 2014). The fact that the salt-bridge interaction identified in E265^7.39^ in the present study was not confirmed for methoxyflavonoids supports this and may provide insight into antagonism by methoxyflavonoids. The results of QSAR analysis indicated that the descriptors *vsurf_S* (interaction surface area) and *wienerPath* (a measure of molecular branching) are crucial for the affinity of polyphenols to T2R. Notably, the coefficient for the descriptor *vsurf_S* (interaction surface area) is negative, whereas the coefficient for the descriptor *wienerPath* (a measure of molecular branching) is positive. These findings indicate that molecules with greater surface area available for interaction and, consequently, more branching are more likely to form robust bonds with T2R46. The fit of 68 known ligands to this model was verified, yielding an R^2^ value of 0.9155, demonstrating the validity of this model for the structure-activity relationship between T2R46 and ligands. According to these results, *vsurf_S* and *wienerPath* were found to be strongly correlated with the binding energy values of bitter compounds, including polyphenols, to T2R46. Furthermore, both the interaction field area and molecular branching tend to increase with molecular weight. This may explain the correlation between molecular weight and binding energy values.

Although this study suggests that many polyphenols show affinity for T2R46, the following points require further investigation. Molecular docking methods, which search for the optimal structure to fit into a receptor pocket, can end up accepting molecules that are larger than the receptor pocket can accommodate. The molecular weights of 68 known T2R46 ligands ranged from 110 to 679, while the molecular weights of the polyphenols that showed low binding energy values in this experiment ranged from 300 to 1900. Further analysis using molecular dynamics techniques may resolve this issue. Furthermore, the expression of the T2R family in taste cells and gastrointestinal secretory cells is intricate, with 4–11 members reported to be expressed in the same cells (Behrens et al., 2007). It is necessary to further investigate what kind of polyphenols can cause bitterness through their interaction with T2R, including *in vivo* experiments. Moreover, *in vitro* discrepancies between studies conducted using T2R-expressing cells in disparate environments can be rectified by the *in silico* molecular docking and QSAR methods employed in this study. Consequently, *in silico* studies will assist in elucidating the mechanism by which polyphenols are perceived as bitter by mammals and exert their health benefits via the secretion of gut hormones.

## 5 Conclusion

The binding affinity of the 490 polyphenols listed in the database to T2R46 was evaluated by *in silico* methods in the course of this experiment. Approximately half of the polyphenols demonstrated affinity with amino acid residues similar to known ligands, with comparable binding energy values. Subsequent QSAR analysis revealed that the interaction surface area and a measure of molecular branching of polyphenol compounds significantly influenced the affinity. The findings indicate that the bitter taste of polyphenols is conveyed to the central nervous system via T2R and that they may possess biological regulatory functions, such as enhancing glucose tolerance, through the action of gut hormones released from gut secretory cells.

## CRediT authorship contribution statement

**Takafumi Shimizu:** Data curation, Investigation, Visualization, Writing – original draft. **Taiki Fushimi:** Investigation, Methodology. **Rio Ohno:** Investigation. **Fujii Yasuyuki:** Formal analysis. **Kenta Aso:** Data curation. **Ursula M. Jacobs:** Writing – review & editing. **Osamu Nureki:** Writing – review & editing. **Yoshitomo Suhara:** Formal analysis, Methodology. **Vittorio Calabrese:** Writing – review & editing. **Naomi Osakabe:** Conceptualization, Funding acquisition, Supervision, Writing – review & editing, All authors discussed the results and commented on the manuscript.

## Declaration of competing interest

The authors declare that they have no known competing financial interests or personal relationships that could have appeared to influence the work reported in this paper.

## Data Availability

No data was used for the research described in the article.

## References

[bib1] Andres-Barquin P.J., Conte C. (2004). Molecular basis of bitter taste: the T2R family of G protein-coupled receptors. Cell Biochem. Biophys..

[bib2] Arts I.C., Hollman P.C. (2005). Polyphenols and disease risk in epidemiologic studies. Am. J. Clin. Nutr..

[bib3] Balaban A.T. (1979). Chemical graphs. Theor. Chim. Acta.

[bib4] Behrens M., Foerster S., Staehler F., Raguse J.-D., Meyerhof W. (2007). Gustatory expression pattern of the human TAS2R bitter receptor gene family reveals a heterogenous population of bitter responsive taste receptor cells. J. Neurosci..

[bib5] Born S., Levit A., Niv M.Y., Meyerhof W., Behrens M. (2013). The human bitter taste receptor TAS2R10 is tailored to accommodate numerous diverse ligands. J. Neurosci..

[bib6] Brockhoff A., Behrens M., Massarotti A., Appendino G., Meyerhof W. (2007). Broad tuning of the human bitter taste receptor hTAS2R46 to various sesquiterpene lactones, clerodane and labdane diterpenoids, strychnine, and denatonium. J. Agric. Food Chem..

[bib7] Brockhoff A., Behrens M., Niv M.Y., Meyerhof W. (2010). Structural requirements of bitter taste receptor activation. Proc. Natl. Acad. Sci. USA.

[bib8] Chandrashekar J., Mueller K.L., Hoon M.A., Adler E., Feng L., Guo W., Zuker C.S., Ryba N.J.P. (2000). T2Rs function as bitter taste receptors. Cell.

[bib9] Chen M.C., Wu S.V., Reeve J.R., Rozengurt E. (2006). Bitter stimuli induce Ca ^2+^ signaling and CCK release in enteroendocrine STC-1 cells: role of L-type voltage-sensitive Ca ^2+^ channels. Am. J. Physiol. Cell Physiol..

[bib10] Chou W.-L. (2021). Therapeutic potential of targeting intestinal bitter taste receptors in diabetes associated with dyslipidemia. Pharmacol. Res..

[bib11] Corbeil C.R., Williams C.I., Labute P. (2012). Variability in docking success rates due to dataset preparation. J. Comput. Aided Mol. Des..

[bib12] Cruciani G., Crivori P., Carrupt P.-A., Testa B. (2000). Molecular fields in quantitative structure–permeation relationships: the VolSurf approach. J. Mol. Struct.: THEOCHEM.

[bib13] Dagan-Wiener A., Di Pizio A., Nissim I., Bahia M.S., Dubovski N., Margulis E., Niv M.Y. (2019). BitterDB: taste ligands and receptors database in 2019. Nucleic Acids Res..

[bib14] Descamps-Solà M., Vilalta A., Jalsevac F., Blay M.T., Rodríguez-Gallego E., Pinent M., Beltrán-Debón R., Terra X., Ardévol A. (2023). Bitter taste receptors along the gastrointestinal tract: comparison between humans and rodents. Front. Nutr..

[bib15] Dotson C.D., Zhang L., Xu H., Shin Y.-K., Vigues S., Ott S.H., Elson A.E.T., Choi H.J., Shaw H., Egan J.M., Mitchell B.D., Li X., Steinle N.I., Munger S.D. (2008). Bitter taste receptors influence glucose homeostasis. PLoS One.

[bib16] Dotson C.D., Vigues S., Steinle N.I., Munger S.D. (2010). T1R and T2R receptors: the modulation of incretin hormones and potential targets for the treatment of type 2 diabetes mellitus. Curr. Opin. Invest. Drugs.

[bib17] D'Urso O., Drago F. (2021). Pharmacological significance of extra-oral taste receptors. Eur. J. Pharmacol..

[bib18] Golbraikh A., Tropsha A. (2002). Beware of q2. J. Mol. Graph. Model..

[bib19] Jalševac F., Terra X., Rodríguez-Gallego E., Beltran-Debón R., Blay M.T., Pinent M., Ardévol A. (2022). The hidden one: what we know about bitter taste receptor 39. Front. Endocrinol..

[bib20] Konovalov D.A., Llewellyn L.E., Vander Heyden Y., Coomans D. (2008). Robust cross-validation of linear regression QSAR models. J. Chem. Inf. Model..

[bib21] Kuroda Y., Ikeda R., Yamazaki T., Ito K., Uda K., Wakabayashi K., Watanabe T. (2016). Activation of human bitter taste receptors by polymethoxylated flavonoids. Biosci. Biotechnol. Biochem..

[bib22] Lang T., Di Pizio A., Risso D., Drayna D., Behrens M. (2023). Activation profile of TAS2R2, the 26th human bitter taste receptor. Mol. Nutr. Food Res..

[bib23] Liszt K.I., Wang Q., Farhadipour M., Segers A., Thijs T., Nys L., Deleus E., Van der Schueren B., Gerner C., Neuditschko B., Ceulemans L.J., Lannoo M., Tack J., Depoortere I. (2022). Human intestinal bitter taste receptors regulate innate immune responses and metabolic regulators in obesity. J. Clin. Invest..

[bib24] Liu K., Jaggupilli A., Premnath D., Chelikani P. (2018). Plasticity of the ligand binding pocket in the bitter taste receptor T2R7. Biochim. Biophys. Acta Biomembr..

[bib25] Lossow K., Hübner S., Roudnitzky N., Slack J.P., Pollastro F., Behrens M., Meyerhof W. (2016). Comprehensive analysis of mouse bitter taste receptors reveals different molecular receptive ranges for orthologous receptors in mice and humans. J. Biol. Chem..

[bib26] Luo M., Ni K., Jin Y., Yu Z., Deng L. (2019). Toward the identification of extra-oral TAS2R agonists as drug agents for muscle relaxation therapies via bioinformatics-aided screening of bitter compounds in traditional Chinese medicine. Front. Physiol..

[bib27] Manach C., Williamson G., Morand C., Scalbert A., Rémésy C. (2005). Bioavailability and bioefficacy of polyphenols in humans. I. Review of 97 bioavailability studies. Am. J. Clin. Nutr..

[bib28] Meyerhof W., Batram C., Kuhn C., Brockhoff A., Chudoba E., Bufe B., Appendino G., Behrens M. (2010). The molecular receptive ranges of human TAS2R bitter taste receptors. Chem. Senses.

[bib29] Molecular Operating Environment (MOE) (2022).

[bib30] Neveu V., Perez-Jimenez J., Vos F., Crespy V., du Chaffaut L., Mennen L., Knox C., Eisner R., Cruz J., Wishart D., Scalbert A. (2010). Phenol-Explorer: an online comprehensive database on polyphenol contents in foods. Database.

[bib31] Osakabe N., Ohmoto M., Shimizu T., Iida N., Fushimi T., Fujii Y., Abe K., Calabrese V. (2024). Gastrointestinal hormone-mediated beneficial bioactivities of bitter polyphenols. Food Biosci..

[bib32] Osakabe N., Shimizu T., Fujii Y., Fushimi T., Calabrese V. (2024). Sensory nutrition and bitterness and astringency of polyphenols. Biomolecules.

[bib33] Pandey K.B., Rizvi S.I. (2009). Plant polyphenols as dietary antioxidants in human health and disease. Oxid. Med. Cell. Longev..

[bib34] Roland W.S.U., Vincken J.P., Gouka R.J., Van Buren L., Gruppen H., Smit G. (2011). Soy isoflavones and other isoflavonoids activate the human bitter taste receptors hTAS2R14 and hTAS2R39. J. Agric. Food Chem..

[bib35] Roland W.S.U., Gouka R.J., Gruppen H., Driesse M., van Buren L., Smit G., Vincken J.-P. (2014). 6-Methoxyflavanones as bitter taste receptor blockers for hTAS2R39. PLoS One.

[bib36] Rozengurt N., Wu S.V., Chen M.C., Huang C., Sternini C., Rozengurt E. (2006). Colocalization of the α-subunit of gustducin with PYY and GLP-1 in L cells of human colon. Am. J. Physiol. Gastrointest. Liver Physiol..

[bib37] Sandal M., Behrens M., Brockhoff A., Musiani F., Giorgetti A., Carloni P., Meyerhof W. (2015). Evidence for a transient additional ligand binding site in the TAS2R46 bitter taste receptor. J. Chem. Theor. Comput..

[bib38] Soares S., Kohl S., Thalmann S., Mateus N., Meyerhof W., De Freitas V. (2013). Different phenolic compounds activate distinct human bitter taste receptors. J. Agric. Food Chem..

[bib39] Soares S., Silva M.S., García-Estevez I., Brás N., Brandão E., Mateus N., De Freitas V., Behrens M., Meyerhof W. (2018). Human bitter taste receptors are activated by different classes of polyphenols. J. Agric. Food Chem..

[bib40] Talmon M., Massara E., Quaregna M., De Battisti M., Boccafoschi F., Lecchi G., Puppo F., Bettega Cajandab M.A., Salamone S., Bovio E., Boldorini R., Riva B., Pollastro F., Fresu L.G. (2023). Bitter taste receptor (TAS2R) 46 in human skeletal muscle: expression and activity. Front. Pharmacol..

[bib41] Tarragon E., Moreno J.J. (2020). Polyphenols and taste 2 receptors. Physiological, pathophysiological and pharmacological implications. Biochem. Pharmacol..

[bib42] Trius-Soler M., Moreno J.J. (2024). Bitter taste receptors: key target to understand the effects of polyphenols on glucose and body weight homeostasis. Pathophysiological and pharmacological implications. Biochem. Pharmacol..

[bib43] Vilar S., Cozza G., Moro S. (2008). Medicinal chemistry and the molecular operating environment (MOE): application of QSAR and molecular docking to drug discovery. Curr. Top. Med. Chem..

[bib44] Vitaku E., Smith D.T., Njardarson J.T. (2014). Analysis of the structural diversity, substitution patterns, and frequency of nitrogen heterocycles among U.S. FDA approved pharmaceuticals. J. Med. Chem..

[bib45] Wang Q., Liszt K.I., Depoortere I. (2020). Extra-oral bitter taste receptors: new targets against obesity?. Peptides.

[bib46] Wiener H. (1947). Structural determination of paraffin boiling points. J. Am. Chem. Soc..

[bib47] Xie C., Wang X., Young R.L., Horowitz M., Rayner C.K., Wu T. (2018). Role of intestinal bitter sensing in enteroendocrine hormone secretion and metabolic control. Front. Endocrinol..

[bib48] Xu W., Wu L., Liu S., Liu X., Cao X., Zhou C., Zhang J., Fu Y., Guo Y., Wu Y., Tan Q., Wang L., Liu J., Jiang L., Fan Z., Pei Y., Yu J., Cheng J., Zhao S. (2022). Structural basis for strychnine activation of human bitter taste receptor TAS2R46. Science.

[bib49] Ziegler F., Steuer A., Di Pizio A., Behrens M. (2023). Physiological activation of human and mouse bitter taste receptors by bile acids. Commun. Biol..

