# Supplementary material for: Verification of the interaction between human bitter taste receptor T2R46 and polyphenols; Computational chemistry approach
Source: Curr Res Food Sci. 2024 Nov 5;9:100914. doi: 10.1016/j.crfs.2024.100914 (PMC11647170; doi:10.1016/j.crfs.2024.100914)
Supplement: Multimedia component 2 [file mmc2.docx]

**Supplementary Methods**

**Details of the structure preparation**

The structural problems were corrected using MOE’s Quick Prep function. The list of structures modified using this function is shown in Table S2. Missing loops are detected when the sequence information is present, but the loop is broken. Such sites are complemented by homology modeling based on the structures of proteins with similar sequences. Gly64-Ser70 is a missing loop structure in the G protein attached to the intracellular part of T2R46, even though sequence information is present (GSGGT). A search for similar sequences at this site yielded Chain A, residue 102-106 of Acetate Kinase (PDB code: 4H0P; Crystal Structure of Acetate Kinase from Cryptococcus neoformans) with 40% homology. Homology modeling was performed based on this structure to complement the loop structure (Fig. S1). Nevertheless, this site is not related to docking because of the cutoff for calculation and does not affect the results.

**Exploration of binding sites for T2R46**

Potential ligand binding sites of T2R46 were predicted using the MOE's Site Finder function. This function automatically detects candidate binding sites on the protein surface based on the protein structure (Fig. S2).

**Details of London ΔG scoring**

The London ΔG scoring follows the equation Eq. (1):

$$\Delta G=c+E_{flex}+\sum_{h-bonds} c_{HB}f_{HB}+\sum_{m-lig} c_{M}f_{M}+\sum_{atoms i} \Delta D_{i} (1)$$

where c is the increase and decrease in rotational and translational entropy, *E_flex_* is the energy due to the loss of ligand flexibility, *c_HB_* is the ideal hydrogen bond energy, *f_HB_* is the imperfection of the hydrogen bond, *c_M_* is the ideal coordination bond energy, *f_M_* is the imperfection of the coordination bond, and *D_i_* is the desolvation energy per atom. This model is employed to score a vast number of ligand placements, with the binding free energy score ΔG are calculated by considering hydrogen bonding, coordination bonding, ligand’s degrees of freedom, and desolvation.

**Details of GBVI/WSA ΔG scoring**

The GBVI/WSA ΔG scoring follows the equation Eq. (2):

$$\Delta G\approx c+\alpha\left[ \frac{2}{3}\left( \Delta E_{coul}+\Delta E_{sol} \right)+E_{vdW}+\beta\Delta SA_{weighted} \right] (2)$$

where *c* is the increase and decrease in rotational and translational entropy, *α*, *β* is the constant determined from the training set in a force field dependent manner, *E_coul_* is the Coulombic electrostatic term, *E_sol_* is the solvation energy according to generalized Born solvation model, *E_vdw_* is the van der Waals term, and *SA_weighted_* is the accessible surface area weighted by solvent exposure. This scoring function is based on the force field, and in this model, the binding free energy is estimated from the ligand’s placement.

**Details of QSAR-Evolution**

This program combines randomly selected descriptors to create many regression equations to form a population, randomly selects two of these equations (but with a bias to select more superior individuals), multiplies them together, and if a superior equation is obtained, replaces by a new generation. Parameters that can be set in QSAR-Evolution include the length of the population's equation, the number of individuals, the mutation probability, the probability of a superior solid leaving offspring to the next generation, the number of generations, and whether the equation is fixed or variable length. Lack of Fit (LOF) was used as the criterion for determining whether an expression of not. It is an evaluation function that automatically penalizes when the number of descriptors used in a model increase.

LOF follows the equation Eq. (3):

$$LOF=\frac{LSE}{\left( 1-\frac{c+dp}{N} \right)^{2}} (3)$$

where *LSE* is the Least square error, *c* is the number of the population excluding constant term, *p* is the number of descriptors included in all populations, *d* is the smoothing parameter, *N* is the number of populations in training set.

In this study, we performed QSAR-Evolution with test set as an option, which allows the algorithm to evolve by considering the score for the test set as well as the LOF, which is an evaluation function. Specifically, all the 490 polyphenols for which descriptors were calculated have been divided in a training set, and in a test set, for the two models’ validation. In particular, tested polyphenols were randomly split into 5 groups using random numbers, with 392 of them as training set (80% of the total tested polyphenols) and 98 as test set (20% of the total tested polyphenols). After that, QSAR-Evolution was performed by varying the calculational conditions, such as the number of descriptors selected for the model, until the obtained model fits both the training and test sets well. Finally, the descriptors in the model equation were substituted with the specific values of the first calculated descriptors of the tested polyphenols, and the fitness between the actual values obtained by molecular docking and the deduced values calculated by the model equation was evaluated.

The model equation is the equation expressed in Eq. (4):

$$Activity=k0+k1\times desc1+k2\times desc2+k3\times desc3\cdots(4)$$

where *Activity* is the response variable (in this case, the binding energy score S), *k#* is the coefficient, *desc#* is the explanatory variable (in this case, descriptors calculated). By focusing on the descriptors selected by the calculation of this model equation, it is possible to derive which elements of the molecule are strongly related to the binding energy score S. Table S8 shows the parameters that can be set in QSAR-Evolution, their descriptions, and the values set in this study.


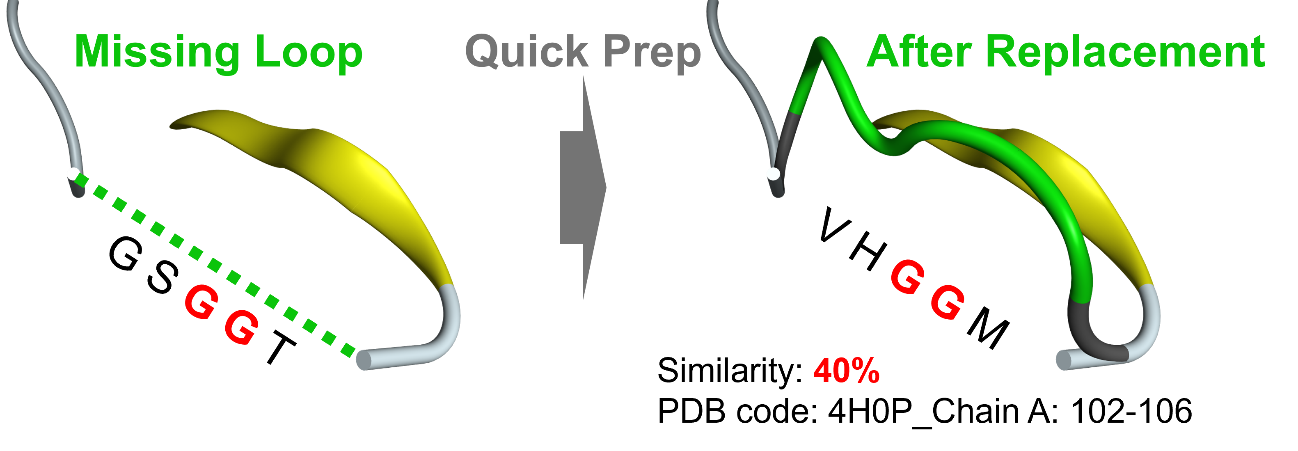


Fig. S1. Loop structure completion with Quick Prep function. Gly64-Ser70 is a missing loop structure in the G protein attached to the intracellular part of T2R46, even though sequence information is present (GSGGT). A search for similar sequences at this site yielded Chain A, residue 102-106 of Acetate Kinase (PDB code: 4H0P; Crystal Structure of Acetate Kinase from Cryptococcus neoformans) with 40% homology. Homology modeling was performed based on this structure to complement the loop structure.

**
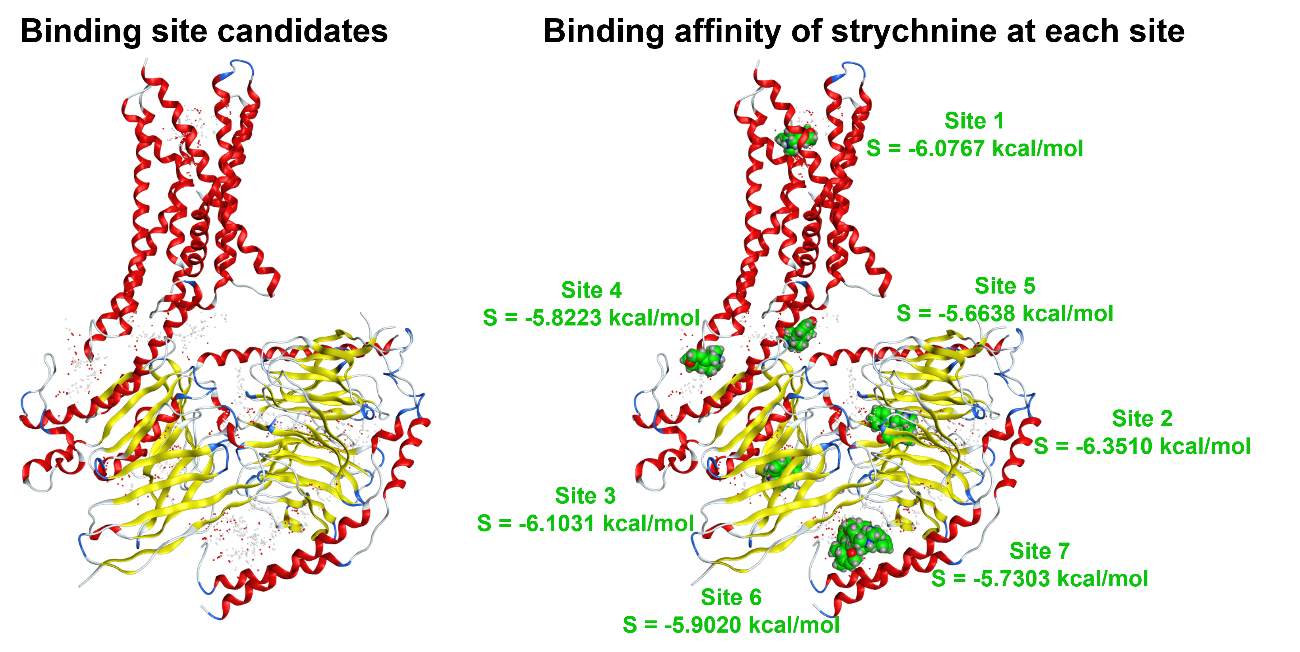
**

Fig. S2. Exploration of binding sites for T2R46. Red spheres: hydrophilic sites, white spheres: hydrophobic sites. Hydrogen-bonding atoms and metal atoms around the spheres were detected, and binding sites were calculated by testing the hydrogen bonding angle and out-of-plane angle. In total, seven candidate ligand binding sites were detected, of which only site 1 was the T2R46 portion of the protein 7XP6.

**
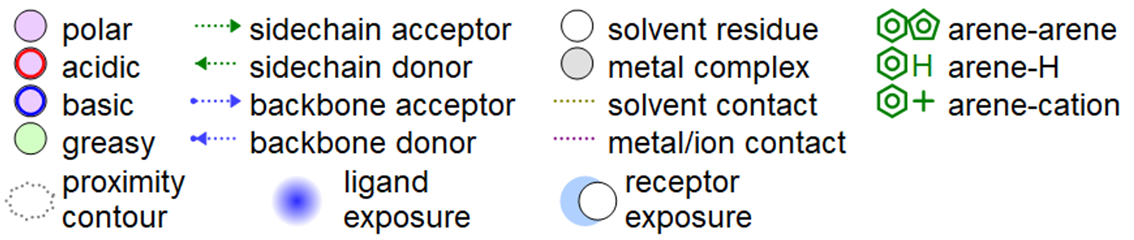
**

Fig. S3. Legend of the 2D ligand interaction. This legend details a two-dimensional view of the ligand-receptor binding structure. Polar: polar amino acid residue, acidic: acidic amino acid residue, basic: basic amino acid residue, greasy: greasy amino acid residue, proximity contour: shape of Receptor pocket, sidechain acceptor: side chain hydrogen bond acceptor, sidechain donor: side chain hydrogen bond donor, backbone acceptor: backbone hydrogen bond acceptor, backbone donor: backbone hydrogen bond donor, ligand exposure: ligand exposure to solvent, receptor exposure: receptor exposure to solvent, arene-arene: arene-arene interaction, arene-H: arene-H interaction arene-cation: arene-cation interaction.


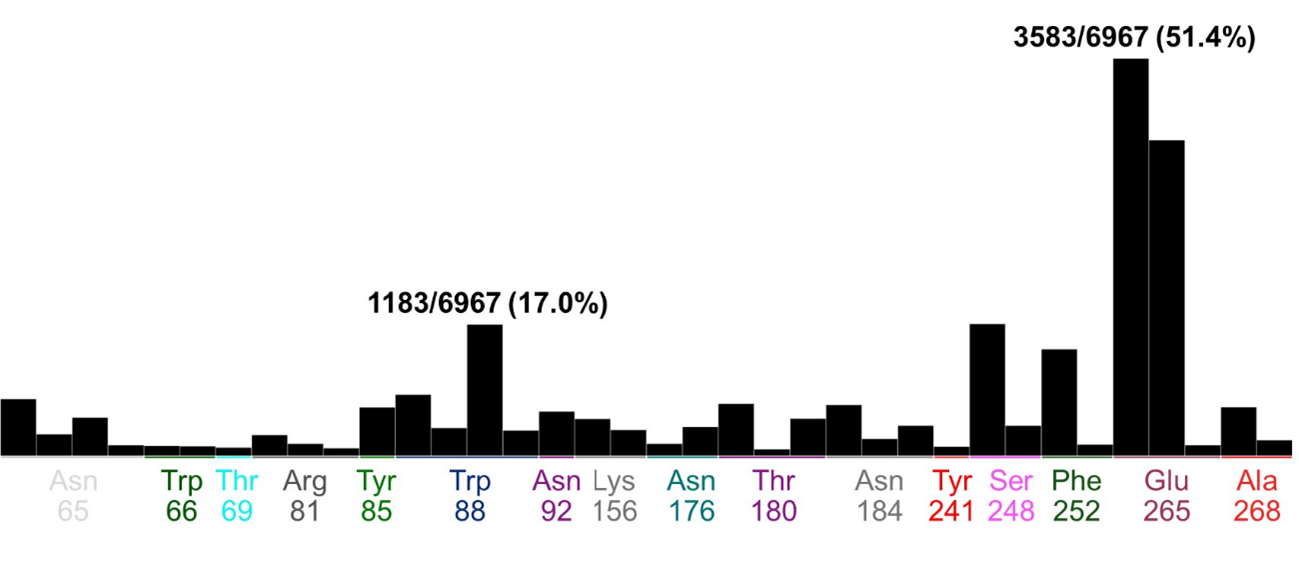


Fig. S4. Population map of the interacting amino acid residues of all results including a total of up to 20 conformations of each polyphenol. E265^7.39^ was the most common interaction in all calculation results (3583/6967; 51.4%). W88^3.32^ was next (1183/6967; 17.0%). In this study, GBVI/WSA ΔG scoring is used to select up to 20 conformations of each compound. This figure shows the results including all conformations.


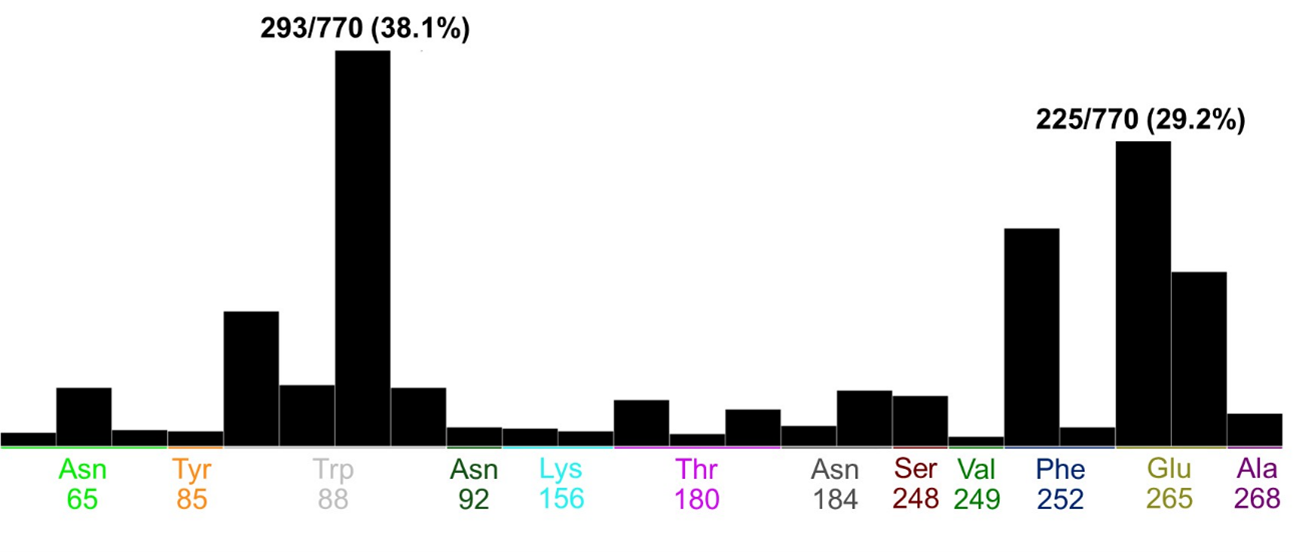


Fig. S5. Population map of the interacting amino acid residues of known T2R46 ligands. W88^3.32^ was the most common interaction in all calculation results (293/770; 38.1%). E265^7.39^ was next (225/770; 29.2%). In this study, GBVI/WSA ΔG scoring is used to select up to 20 conformations of each compound. This figure shows the results including all conformations.

Table S2. The list of structures that have been modified using Quick Prep function.

| **Residue** | **Residue Number** | **Type of Problems** | **Details** |
| --- | --- | --- | --- |
| - | - | Hcount | Incorrect number of hydrogens |
| - | - | Charge | System is not charged |
| Gly | 64 | Break | Build 5-loop (Gly64-Ser70) |
| Ser | 70 | Library | Ambigious residue name (SER) |
| Phe | 249 | Termini | Charge C termius |
| Glu | 3 | Termini | Cap N terminus, delete 2 residue outgap |
| Glu | 3 | Topology | Incorrect Hybridization |
| Thr | 6 | Termini | Cap N terminus, delete 5 residue outgap |
| Thr | 6 | Library | Ambigious residue name (THR) |

This function automatically diagnoses protein structures and corrects problems. Hcount: number of hydrogen atoms, Charge: partial charges, Break: sequence/structure mismatches, Library: structures not found in the amino acid library, Termini: structural problems at the N- and C-terminal, Topology: topological problems present in the residues.

Table S5. Details of interaction shown in Fig. 6.

| Interaction # | Residue | Interaction type | %abund |
| --- | --- | --- | --- |
| 1 | Val 61 | BkDon1 | 1.1033113 |
| 2 | Asn65 | ChDon1 | 5.2980574 |
| 3 | Asn65 | ChDon2 | 2.4283664 |
| 4 | Asn65 | ChAcc1 | 1.7663797 |
| 5 | Trp 66 | Arene1 | 1.1033113 |
| 6 | Arg 81 | ChAcc1 | 3.5316777 |
| 7 | Arg 81 | ChAcc2 | 1.1033113 |
| 8 | Arg 81 | BkDon1 | 1.3239735 |
| 9 | Tyr 85 | Arene1 | 3.9730022 |
| 10 | Trp 88 | ChAcc1 | 13.245143 |
| 11 | Trp 88 | ChAcc2 | 7.5057616 |
| 12 | Trp 88 | Arene1 | 46.357461 |
| 13 | Trp 88 | Arene2 | 1.9870419 |
| 14 | Asn 92 | ChDon1 | 11.258102 |
| 15 | Lys 156 | ChAcc1 | 2.6490287 |
| 16 | Lys 156 | ChAcc2 | 1.9870419 |
| 17 | Asn 176 | ChDon1 | 2.6490287 |
| 18 | Asn 176 | BkDon1 | 2.8696909 |
| 19 | Thr 180 | ChDon1 | 4.4154084 |
| 20 | Thr 180 | Arene1 | 2.4283664 |
| 21 | Asn 184 | ChDon1 | 7.0644371 |
| 22 | Asn 184 | ChDon2 | 1.9870419 |
| 23 | Asn 184 | ChAcc1 | 5.9600442 |
| 24 | Asn 184 | ChAcc2 | 1.5457174 |
| 25 | Tyr 241 | ChDon1 | 1.3239735 |
| 26 | Ser 248 | ChDon1 | 15.011523 |
| 27 | Ser 248 | ChDon2 | 4.1947461 |
| 28 | Ser 248 | ChAcc1 | 1.1033113 |
| 29 | Phe 252 | Arene1 | 22.074879 |
| 30 | Glu 265 | ChDon1 | 55.187196 |
| 31 | Glu 265 | ChDon2 | 47.461854 |
| 32 | Ala 268 | BkDon1 | 11.037439 |
| 33 | Ala 268 | BkDon2 | 5.7393819 |
| 34 | Ala 268 | Arene1 | 1.9870419 |

This table shows the percentages and the details of each interaction shown in Fig. 6; population map of the interacting amino acid residues after extracting the results of polyphenols with the lowest binding energy score. BkDon: backbone hydrogen bond donor interaction, ChDon: sidechain hydrogen bond donor interaction, ChAcc: sidechain hydrogen bond acceptor interaction, Arene: arene interaction.

Table S6. Details of interaction shown in Fig. S4.

| Interaction # | Residue | Interaction Type | %abund |
| --- | --- | --- | --- |
| 1 | Asn 65 | ChDon1 | 7.449585 |
| 2 | Asn 65 | ChDon2 | 2.957097 |
| 3 | Asn 65 | ChAcc1 | 5.066253 |
| 4 | Asn 65 | ChAcc2 | 1.550102 |
| 5 | Trp 66 | ChAcc1 | 1.449794 |
| 6 | Trp 66 | Arene1 | 1.406995 |
| 7 | Thr 69 | ChDon1 | <1.000 |
| 8 | Arg 81 | ChAcc1 | 2.842077 |
| 9 | Arg 81 | ChAcc2 | 1.737345 |
| 10 | Arg 81 | BkDon1 | <1.000 |
| 11 | Tyr 85 | Arene1 | 6.37294 |
| 12 | Trp 88 | ChAcc1 | 7.995264 |
| 13 | Trp 88 | ChAcc2 | 3.760904 |
| 14 | Trp 88 | Arene1 | 16.96553 |
| 15 | Trp 88 | Arene2 | 3.430554 |
| 16 | Asn 92 | ChDon1 | 5.856685 |
| 17 | Lys 156 | ChAcc1 | 4.89506 |
| 18 | Lys 156 | ChAcc2 | 3.502776 |
| 19 | Asn 176 | ChDon1 | 1.737345 |
| 20 | Asn 176 | BkDon1 | 3.889299 |
| 21 | Thr 180 | ChDon1 | 6.831684 |
| 22 | Thr 180 | Any | <1.000 |
| 23 | Thr 180 | Arene1 | 4.937858 |
| 24 | Asn 184 | ChDon1 | 6.703289 |
| 25 | Asn 184 | ChDon2 | 2.353908 |
| 26 | Asn 184 | ChAcc1 | 4.033743 |
| 27 | Tyr 241 | ChDon1 | 1.377571 |
| 28 | Ser 248 | ChDon1 | 17.02304 |
| 29 | Ser 248 | ChDon2 | 4.033743 |
| 30 | Phe 252 | Arene1 | 13.80781 |
| 31 | Phe 252 | Arene2 | 1.622324 |
| 32 | Glu 265 | ChDon1 | 51.42755 |
| 33 | Glu 265 | ChDon2 | 40.53404 |
| 34 | Glu 265 | Ionic1 | 1.53539 |
| 35 | Ala 268 | BkDon1 | 6.401026 |
| 36 | Ala 268 | BkDon2 | 2.19609 |

This table shows the percentages and the details of each interaction shown in Fig. S4; population map of the interacting amino acid residues including all conformations. BkDon: backbone hydrogen bond donor interaction, ChDon: sidechain hydrogen bond donor interaction, ChAcc: sidechain hydrogen bond acceptor interaction, Arene: arene interaction, Any: any interaction, Ionic: ionic interaction.

Table S8. Computational conditions for QSAR-Evolution.

| Parameter | Description | Value |
| --- | --- | --- |
| Length | The number of descriptors included in one expression. | 2 |
| Population | The number of model expressions included in the population. | 1000 |
| Operator Density | The percentage of descriptors to which the operator applies. | 4 |
| Generation | The number of steps to try to change generations. | 500000 |
| Mutation Probability | At 0.5, once every two steps, one of the descriptors could replace one not included in the parents. | 0.5 |
| Eugenic Factor | The ratio of the probability that the best and worst formulas in the population are involved in alteration of generations. | 100 |
| Auto Termination | If the value of the evaluation function does not change for all the expressions in the population during the number of steps specified here, the evolution is terminated. | 1000 |

Table S9. Details of interaction shown in Fig. 9.

| Interaction # | Residue | Interaction Type | %abund |
| --- | --- | --- | --- |
| 1 | Asn 65 | ChAcc2 | 4.444178 |
| 2 | Trp 66 | ChAcc1 | 2.222844 |
| 3 | Trp 66 | ChAcc2 | 2.222844 |
| 4 | Tyr 85 | BkAcc1 | 2.222844 |
| 5 | Tyr 85 | Arene1 | 4.444178 |
| 6 | Trp 88 | Arene1 | 26.66658 |
| 7 | Trp 88 | ChAcc2 | 15.55538 |
| 8 | Trp 88 | ChAcc1 | 26.66658 |
| 9 | Trp 88 | Arene2 | 2.222844 |
| 10 | Lys 156 | ChDon1 | 8.888356 |
| 11 | Lys 156 | ChAcc1 | 2.222844 |
| 12 | Lys 156 | ChAcc2 | 2.222844 |
| 13 | Thr 180 | Arene1 | 2.222844 |
| 14 | Thr 180 | ChDon2 | 2.222844 |
| 15 | Thr 180 | ChDon1 | 2.222844 |
| 16 | Asn 184 | ChDon1 | 6.667022 |
| 17 | Asn 184 | ChAcc1 | 11.1112 |
| 18 | Ser 248 | ChDon1 | 4.444178 |
| 19 | Phe 252 | Arene1 | 11.1112 |
| 20 | Phe 252 | Arene2 | 2.222844 |
| 21 | Glu 265 | ChDon1 | 24.44373 |
| 22 | Glu 265 | ChDon2 | 13.33404 |
| 23 | Ala 268 | BkDon1 | 4.444178 |
| 24 | Phe 269 | BkAcc1 | 2.222844 |

This table shows the percentages and the details of each interaction shown in Fig. 9; population map of the interacting amino acid residues after extracting the results of known ligands with the lowest binding energy score. BkDon: backbone hydrogen bond donor interaction, BkAcc: backbone hydrogen bond acceptor interaction, ChDon: sidechain hydrogen bond donor interaction, ChAcc: sidechain hydrogen bond acceptor interaction, Arene: arene interaction.

Table S10. Details of interaction shown in Fig. S5.

| Interaction # | Residue | Interaction Type | %abund |
| --- | --- | --- | --- |
| 1 | Asn 65 | ChDon1 | 1.4288 |
| 2 | Asn 65 | ChAcc1 | 5.7136 |
| 3 | Asn 65 | ChAcc2 | 1.688 |
| 4 | Tyr 85 | Arene1 | 1.5584 |
| 5 | Trp 88 | ChAcc1 | 12.9872 |
| 6 | Trp 88 | ChAcc2 | 5.9744 |
| 7 | Trp 88 | Arene1 | 38.0512 |
| 8 | Trp 88 | Arene2 | 5.7136 |
| 9 | Asn 92 | ChDon1 | 1.9488 |
| 10 | Lys 156 | ChAcc1 | 1.8176 |
| 11 | Lys 156 | ChAcc2 | 1.5584 |
| 12 | Thr 180 | ChDon1 | 4.5456 |
| 13 | Thr 180 | ChDon2 | 1.2992 |
| 14 | Thr 180 | Arene1 | 3.6368 |
| 15 | Asn 184 | ChDon1 | 2.0784 |
| 16 | Asn 184 | ChAcc1 | 5.4544 |
| 17 | Ser 248 | ChDon1 | 4.9344 |
| 18 | Val 249 | BkAcc1 | 1.0384 |
| 19 | Phe 252 | Arene1 | 20.9088 |
| 20 | Phe 252 | Arene2 | 1.9488 |
| 21 | Glu 265 | ChDon1 | 29.2208 |
| 22 | Glu 265 | ChDon2 | 16.7536 |
| 23 | Ala 268 | BkDon1 | 3.2464 |

This table shows the percentages and the details of each interaction shown in Fig. S5; population map of the interacting amino acid residues including all conformations. BkDon: backbone hydrogen bond donor interaction, BkAcc: backbone hydrogen bond acceptor interaction, ChDon: sidechain hydrogen bond donor interaction, ChAcc: sidechain hydrogen bond acceptor interaction, Arene: arene interaction.
